# Women's empowerment, maternal depression, and stress: Evidence from rural Burkina Faso

**DOI:** 10.1016/j.ssmmh.2022.100160

**Published:** 2022-12

**Authors:** Jessica Leight, Abdoulaye Pedehombga, Rasmané Ganaba, Aulo Gelli

**Affiliations:** a International Food Policy Research Institute (IFPRI), USA; b AfricSanté, Burkina Faso

**Keywords:** Mental health, Empowerment, Stress, Depression

## Abstract

**Objective:**

Though there is a wide array of evidence that women's empowerment is associated with more positive health and nutritional outcomes for women and children, evidence around the relationship with mental health or subjective well-being remains relatively limited. The objective of this paper is to explore this relationship in longitudinal data from rural Burkina Faso.

**Methods:**

We analyze the association between empowerment measured using the project-level Women's Empowerment in Agriculture Index (pro-WEAI), and two additional outcomes of interest: stress (measured using the SRQ-20) and maternal depression (measured using the Edinburgh scale for post-partum depression). The analysis employs both cross-sectional specifications and panel specifications conditional on individual fixed effects.

**Results:**

We find evidence of substantial negative correlations between the empowerment score and maternal stress and depression measured using both continuous and binary variables. This relationship seems to be particularly driven by self-efficacy and respect among household members, where higher scores have negative associations with depression and stress that are both large in magnitude and precisely estimated.

**Conclusion:**

Enhanced mental health may be another channel for a positive effect of empowerment on women's welfare.

## 1 Introduction

There is growing interest in both research and policy settings in identifying strategies to enhance women's empowerment; empowering women is both an important goal in and of itself, and a step that can lead to a range of other positive effects for women and children. A large body of literature has found that women who are more empowered using various definitions utilize more health care for themselves as well as their children, and may achieve better health care outcomes (Allendorf, 2007; Bliznashka et al., 2021; Furuta and Salway, 2006; Lee et al., 2008; Lépine and Strobl, 2013; Maitra, 2004; Mistry et al., 2009). Women's empowerment is also associated with better nutritional outcomes, both at the household level and the child level (Cunningham et al., 2015; Malapit et al., 2015; Ruel et al., 2018; Sraboni et al., 2014).

However, there is relatively limited evidence to date around the effect of empowerment on women's mental health or subjective well-being. There are a number of channels through which empowerment could shift well-being, and these channels generally, though not uniformly, suggest a positive relationship. For example, women who are more empowered may enjoy more social support, and may have more access to economic or other resources inside or outside the household; both social support and access to economic resources are correlated with better mental health (Myer et al., 2008; Patel et al., 2006; Rungreangkulkij et al., 2019). Empirical evidence using various definitions of both empowerment and mental health has suggested a positive association (empowerment is associated with better mental health) in cross-sectional analyses in Senegal, India, Laos, and Egypt (Fielding and Lepine, 2017; Scott et al., 2020; Yoshikawa and Kamiya, 2019; Yount et al., 2014). In rural Burkina Faso, the site of this analysis, there is no prior research on the relationship between empowerment and mental health, and very limited literature around mental health more broadly (Filippi et al., 2007; Lokubal et al., 2021; Prado et al., 2017, 2019).

The objective of this paper is to provide new evidence about the relationship between women's empowerment, measured using the project-level Women's Empowerment in Agriculture Index (pro-WEAI), and women's mental health, as measured using the Self-Reported Questionnaire (SRQ-20) stress scale and the Edinburgh index of post-partum depression, in a large sample from rural Burkina Faso. The data was collected between 2017 and 2020 as part of a large-scale randomized trial called SELEVER (Gelli et al., 2017), but we focus here on estimating cross-sectional and longitudinal relationships between empowerment and well-being for a sample of approximately 1500 women. The empirical strategy is simple, and entails the exploration of cross-sectional associations at baseline between the continuous empowerment score and a binary variable for empowered and the well-being variables of interest. In addition, the same variables are analyzed in a panel specification, conditional on individual fixed effects; this specification effectively controls for any time-invariant unobservable characteristics that may be correlated with both empowerment and mental health.

Our analysis thus addresses several notable gaps in the literature. We conduct the first longitudinal analysis of the relationship between empowerment and mental health, the first analysis using detailed data capturing various facets of empowerment, and the first analysis in Burkina Faso. Drawing on the richness of the pro-WEAI measures of empowerment, along with detailed socioeconomic data and multiple measures of mental health, we are able to provide a uniquely nuanced account of how multiple facets of empowerment shape well-being in this setting. Our findings are consistent with the earlier literature in suggesting a positive association, and particularly highlight that self-efficacy and respect among household members may be the primary channels for this association.

## 2 Conceptual framework

In our analysis, empowerment will be defined following the conceptualization employed by the project-level Women's Empowerment in Agriculture (pro-WEAI) index, the index used to measure empowerment. Based on this conceptualization, empowerment is envisioned as a process in which women can claim resources that enhance their agency, or the ability to make strategic choices that enable them to achieve their own goals or collective goals (Kabeer, 1999). The pro-WEAI index has been previously developed and validated in a range of contexts (Malapit et al., 2019).

**Fig. 1:**
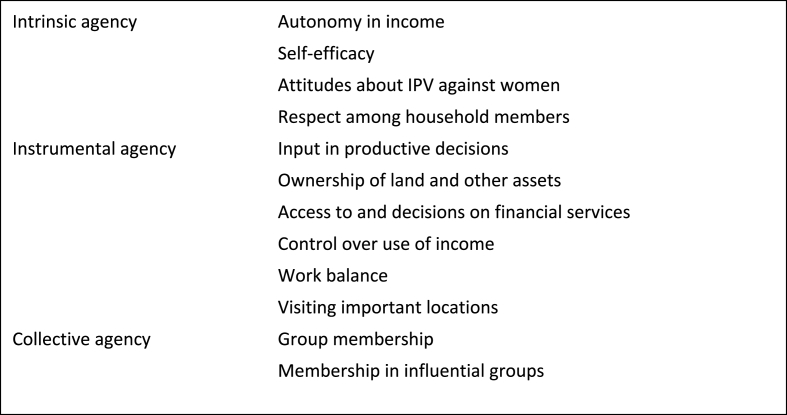
Pro-WEAI indicators.

There are a number of channels through which women's empowerment could affect mental health and well-being. Anchoring again to the conceptualization employed by the pro-WEAI index, the index encompasses three domains: intrinsic agency (“power within”), instrumental agency (“power to”), and collective agency (“power with”). These domains are themselves comprised by 12 specific indicators or subdomains, as summarized in Fig. 1. We use these indicators to structure the conceptual framework, seeking to understand the following question: what evidence is there for a relationship between empowerment and mental health, operating through this specific indicator? Note that this analysis does not entail an assumption that a woman who is characterized by at least partial empowerment is empowered in a particular subdomain, as of course women may, and do, experience empowerment in some subdomains and not others. Our goal is to understand how empowerment in a particular subdomain, if observed, could shape a woman's mental health.

These relationships between multiple facets of empowerment and mental health been explored and documented in the existing literature across multiple disciplines, and here, we analyze the literature focused on low or middle-income countries (LMICs). We do not limit this discussion to analyses drawing on data from Burkina Faso given that there is, unfortunately, no relevant literature from Burkina specifically; similarly, only a small number of extant studies examine the relationships of interest in sub-Saharan Africa, given the paucity of large-scale quantitative studies analyzing mental health in the region. For these reasons, we draw on data from a wide range of developing country contexts.^1^

This literature is primarily associational, a key limitation, and associations that are observed in other regions may not necessarily apply in rural Burkina Faso. In addition, the relationship between empowerment and maternal health is by no means assumed to be positive (though the prior evidence from developing countries, as noted in the introduction, does generally suggest a positive relationship). Particularly in a context such as Burkina Faso where concepts of empowerment may be very different vis-à-vis a high-income or Western context, the relationship could be negative, and in elucidating the channels of interest, we will note specific channels that could be consistent with this negative association.

### 2.1 Participation in household decision-making

Women who are more empowered may be able to participate more actively in household decision-making. This relates to the first pro-WEAI indicator in the domain of intrinsic agency (autonomy in income), as well as the fifth pro-WEAI indicator in the domain of instrumental agency (input in productive decisions).

There is some evidence that active participation in decision-making may be correlated with mental health, though the pattern is mixed. In an analysis in Thailand, decision-making power was not significantly associated with depressive symptoms in a multivariate analysis (Rungreangkulkij et al., 2019). However, among a sample of women in Uganda, a higher level of decision-making power as it relates to sexual relationships was predictive of enhanced mental health (Hatcher et al., 2012); and among samples of postpartum women in both Ethiopia and Taiwan, absence of empowerment in domestic decision-making was associated with a higher level of postpartum depression (Abebe et al., 2019; Chen and Chien, 2020). In Pakistan, decision-making authority was associated with a significantly higher level of happiness for women (Mubashir Ali and Ul Haq, 2006). In a sample of older Mexican adults, an imbalance of decision-making power within the spousal relationship was associated with a higher prevalence of depressive symptoms (Saenz and Rote, 2019). In South Africa, making decisions alone (without one's partner) was associated with a higher level of depressive symptoms (Hamad et al., 2008).

### 2.2 Higher self-efficacy

Women who are more empowered may be characterized by higher self-efficacy. This is the second pro-WEAI indicator in the domain of intrinsic agency, self-efficacy. While the relationship between self-efficacy and mental health is doubtless highly context specific, some evidence has suggested this relationship may be positive: higher self-efficacy is associated with higher levels of well-being (Andersson et al., 2014; Huang et al., 2021; Parto, 2011). Measuring concepts such as self-efficacy across very different country contexts can also be challenging, but a study across 25 countries suggested that the general self-efficacy scale had stable psychometric properties throughout the sample (Scholz et al., 2002).

In sub-Saharan Africa, there is little widespread evidence on the relationship between self-efficacy and other psychosocial or health outcomes, though some research has explored more specific measures of self-efficacy linked to contraceptive use or reproductive decision-making (Closson et al., 2018; Whiting-Collins et al., 2020). There is some, albeit minimal, evidence from other LMICs. A multicountry study in five countries including three high-income and middle-income countries (Poland, Germany, and Costa Rica) found that general self-efficacy was associated with lower levels of depression and anxiety (Luszczynska et al., 2005). In addition, an analysis of Indian and Iranian university students attending a university in India found that low self-efficacy was associated with depression (Ghaderi and Rangaiah, 2011).

### 2.3 Reduced intimate partner violence

Women who are more empowered may experience lower levels of intimate partner violence. That being said, the reverse relationship can also be observed in which women who are more empowered in other domains experience higher intimate partner violence due to male backlash (Angelucci and Heath, 2020; Bulte and Lensink, 2019). This relates to the third pro-WEAI indicator within the domain of intrinsic agency, attitudes around intimate partner violence.

Women who experience lower levels of IPV are likely to experience substantially enhanced mental health, as there is strong evidence that higher IPV is associated with higher levels of depression and lower well-being (Campbell, 2002; Ishida et al., 2010; Mahenge et al., 2013; Meekers et al., 2013; Oram et al., 2017; Stephenson et al., 2013; Vizcarra et al., 2004).

### 2.4 More respectful relationships with family members

Women who are more empowered may have more respectful and mutually beneficial members with spouses and family members. This relates to the fourth pro-WEAI indicator within the domain of intrinsic agency, respect among household members. Evidence suggests marital quality or marital conflict was an important determinant of women's experience of depression in Bangladesh, Pakistan, and Tanzania (Gausia et al., 2009; Hamad et al., 2008; Kaaya et al., 2010). In addition, the ability to exert control over or participate in decisions around intimate relationships and sexual behavior – measured using the Sexual Relationship Power Scale – has been shown to be correlated with a lower probability of depressive symptoms in Uganda (Hatcher et al., 2012) and South Africa (Conroy et al., 2016).

### 2.5 Enhanced economic welfare

Women who are more empowered may be more likely to own or jointly own assets, and/or may have more access to financial resources and income. This relates to the sixth, seventh and eighth pro-WEAI indicators within the domain of instrumental agency (ownership of land and other assets, access to and decisions on financial services, and control over use of income).

The relationship between economic status (poverty and asset ownership) and mental health is complex and appears to be context-specific (Baird et al., 2013; Cole and Tembo, 2011; Das et al., 2007; Hadley et al., 2008; Lund et al., 2011; Patel and Kleinman, 2003). Higher economic status was associated with higher well-being and/or a lower prevalence of mental health challenges among a sample of rural women in India (Patel et al., 2006), in samples of adults in South Africa (Hamad et al., 2008; Myer et al., 2008), and in a sample of older adults in Thailand (Rungreangkulkij et al., 2019).

### 2.6 More manageable workload

Women who are more empowered may experience a more manageable workload. This relates to the ninth pro-WEAI indicator within the domain of instrumental agency, work balance. While there is limited evidence in the literature on the relationship between time use and well-being or mental health in low-income countries, some evidence suggests that the length and intensity of work time may be an important determinant of women's well-being, and that excessive engagement in work has detrimental effects on self-reported well-being (Floro and Pichetpongsa, 2010; Floro and Seymour, 2016; Richardson et al., 2017).

### 2.7 More access to health or community resources outside the household

Women who are more empowered may also be more likely to access resources outside the household, including health facilities or health services as well as other community resources that could be beneficial for their mental health (e.g. religious or cultural associations or events; or markets or other sites where they can access goods and services). This relates to the tenth pro-WEAI indicator within the domain of instrumental agency, visiting important locations. In general, access to mental health services in sub-Saharan Africa is quite limited (Jenkins et al., 2010; Skeen et al., 2010), and thus the evidence base around the relationship between such access and self-reported well-being seems minimal.

### 2.8 More social connections outside the household

Women who are more empowered may have stronger social connections outside their household (with neighbors, community members, or extended family members), or may participate more in local associational life. This relates to the eleventh and twelfth pro-WEAI indicators within the domain of collective agency, group membership and membership in influential groups. Evidence suggests that social support is generally associated with better mental health across a range of populations. One study from southern Africa showed that women who are members of community groups (predominantly church groups) in Zambia and South Africa reported higher self-rated well-being (Thomas, 2006), and a second study in South Africa suggested low social capital and social support were associated with higher psychological distress (Myer et al., 2008). Similarly, low social support was a risk factor for depressive symptoms in a sample of older Thai adults (Rungreangkulkij et al., 2019), and evidence from Pakistan suggested that perceived social support was associated with a reduced risk of depression (Qadir et al., 2013). Low social support was also identified as a risk factor for postpartum depression in Uganda, Nigeria, and Pakistan (Adewuya et al., 2007; Atuhaire et al., 2021; Rahman et al., 2003; Wubetu et al., 2020).

## 3 Data and empirical strategy

### 3.1 Context

This paper draws on data from a large-scale randomized controlled trial conducted in rural Burkina Faso, SELEVER (Soutenir l’Exploitation Familiale pour Lancer l’Élevage des Volailles et Valoriser l’Économie Rurale). SELEVER was a five-year program implemented by Tanager in conjunction with a range of partners, with the objective of increasing poultry production and improving the nutritional status of women and children in the Centre Ouest, Hauts-Bassins and Boucle de Mouhoun regions (Gelli et al., 2017). SELEVER was also evaluated in a large-scale randomized controlled trial conducted by IFPRI between 2017 and 2020, and the data from this trial (described in more detail in the next section) will be used to inform this analysis.

Available demographic and health indicators from Burkina Faso are suggestive of meaningful challenges linked to women's well-being. Data from the most recent Demographic and Health Survey conducted in 2010 indicated that 76% of women report no or minimal literacy; the total fertility rate is high, at six children per woman on average. The median age at first marriage is slightly under 18, and the median age at first birth is slightly younger than 20. However, large-scale population-based data reporting stress, post-partum depression or other indices of women's well-being in Burkina Faso is largely absent. Two existing related studies analyze the relationship between relationship satisfaction and postpartum health-related quality of life among women in Burkina Faso (Lokubal et al., 2021), as well as the effect of severe obstetric complications on a range of outcomes, including depression and anxiety (Filippi et al., 2007). However, these are smaller and context-specific samples, and the first paper does not report any mental health measure, relying on health-related quality of life.

### 3.2 Intervention and data

SELEVER was designed as a multifaceted intervention seeking to increase poultry production and improve the nutritional status of women and children among poor rural households (Gelli et al., 2017). It included three key elements: promotion of poultry production via training for producer households and enhanced access to veterinary inputs and training; behavioral change communication around nutrition and health designed to promote improved diets for women and children; and community-level sensitization around women's economic empowerment. Importantly, there was no transfer of assets or provision of subsidized or free inputs. Randomization was conducted at the commune level, and the intervention was targeted primarily to existing associations of poultry producers that served as the platform for intervention activities. More details are provided in the supplementary material.

The sample for the SELEVER evaluation included 60 communes (rural and peri-urban) within the three targeted regions, selected randomly from a group of 79 communes in identified as eligible for scale-up based on the following criteria: they had not previously been exposed to SELEVER pilot programming, they were designated as rural or peri-urban in the national census, and they were accessible by road year round. In sampled communities, households with a woman 15–35 years of age and a child aged 2–4 years living together were eligible for inclusion. A full household census was conducted to identify eligible households, and 15 households were then randomly selected for inclusion. This yields a target baseline sample of 1800 households (60 communes, 120 villages). Ultimately, data was collected in only 119 villages; one village was omitted due to a failure to correctly identify the community in the field.

The SELEVER surveys were wide-ranging and administered to both women (the mother of the index child aged 2–4 years) and men (usually the head of household, or alternatively the spouse of the mother of the index child). The surveys collected data on household economic status and engagement in productive activities (particularly poultry production), men's and women's knowledge and behaviors around nutrition and hygiene, women's well-being, diets for the index women and child, and empowerment. Anthropometric measurement was also conducted.

For the purposes of this analysis, we focus on the sample of women who were the mothers of the index children and who were surveyed in the two key modules analyzed here -- women's empowerment, and maternal well-being (stress and post-partum depression). The women's empowerment module was administered to the woman who was most knowledgeable about poultry production, and accordingly this subsample includes women who were the mother of a young child and also relatively active in livestock production. This yields a sample of 1203 women at baseline; for the panel analysis, we restrict further to women who were observed at both baseline and endline, yielding a sample of 1110 women in the panel, or 2220 observations. (All 119 sampled villages are represented in the analysis at baseline, and 118 villages are represented in the panel analysis.) The household-level attrition rate from baseline to endline was only 7%.

Ethical approval was provided by the Institutional Research Board at the International Food Policy Research Institute and the Comité Éthique pour la Recherche en Santé MS/MRSI in Burkina Faso.

### 3.3 Primary variables of interest

As previously noted, empowerment is measured using the pro-WEAI index. The index is constituted by 12 equally weighted indicators, and an individual is considered to be empowered if she or he is adequate in at least 75% (nine out of 12) indicators, a threshold that was defined when the pro-WEAI index was developed (Malapit et al., 2019). Though the cutoff of eight was not based on any specific quantitative criteria, it was informed by the qualitative judgment of the research team developing the index as well as feedback from projects using the index; previous evidence also demonstrates that in the five survey samples used for validation, there was no shift in cross-sample rankings of empowerment for any cut-off between five and nine, and minimal shifts for any cut-off between four and 11 (Malapit et al., 2019). In this survey, the pro-WEAI was administered to both men and women in each household, but our analysis draws only on the data collected from women.

Women's mental health is measured using two indices. The first is the self-report questionnaire SRQ-20 (World Health Organization, 1994), an index that can be used to capture depressive symptoms but is often interpreted as a scale of maternal stress (Mobarak et al., 2000; Olney et al., 2019; Ziaei et al., 2016). The SRQ-20 has not been validated specifically in Burkina Faso, but has been used in other studies in the country (Prado et al., 2017, 2019) and has been validated in sub-Saharan Africa (Sweetland et al., 2014). The second is the Edinburgh postnatal depression scale, a widely employed scale that has also been validated in a number of sub-Saharan African contexts, though not in Burkina Faso specifically (Cox et al., 1987; Tsai et al., 2013). The Cronbach's alpha for the SRQ-20 is 0.82 and for the Edinburgh scale is 0.88 in this sample, suggesting a high level of internal consistency for both indices.

For the stress scale, we analyze the continuous variable (ranging from zero to 20) as well as a binary variable for high maternal stress. A wide range of thresholds have been employed in the literature in the region (Sweetland et al., 2014). Only two previous studies were conducted in West Africa (in Ghana and Nigeria), and we employ the cutoff of five used in Nigeria (Abiodun, 1988), as the sensitivity and specificity of this variable in the population studied is reported to be higher vis-à-vis the comparable scale analyzed in Ghana (Weobong et al., 2009). For the Edinburgh scale, we employ both the continuous variable capturing the magnitude of depressive symptoms (ranging from 0 to 27) and a binary variable using the validated threshold for postpartum depression, a symptom score greater than or equal to 12.

One important point to note in interpreting the results employing the Edinburgh scale is that this is not generally a population that is immediately postpartum or even within a year of their most recent delivery, a commonly employed period for analysis of perinatal depression (Gaynes et al., 2005). At baseline, the index child was between two and four, and 25% of women reported another child aged 12 months or younger. By endline, the index child was between five and seven, and around 21% of women reported another child aged 12 months or younger. A systematic review of literature analyzing postpartum depression beyond the immediate postpartum period found studies including analysis of the Edinburgh scale up to two and a half years following delivery (Goodman, 2004), and some papers have used the same variable to analyze women up to four years postpartum (Josefsson and Sydsjö, 2007). The empirical analysis will also explore the relevance of the age of the youngest child as a variable that may be predictive of maternal mental health.

The choice of mental health indicators measured in the trial, and analyzed here, was substantially informed by the broader context of the trial. Given that the target population was uniformly mothers of infants and young children, measuring depression using the Edinburgh scale was deemed appropriate for their life stage. The choice of stress as a second mental health measure reflected the hypothesis that women's engagement in new productive activities (as promoted by SELEVER) and/or the expansion of household poultry production could lead to increased stress, given the potential for an increased time commitment, and the uncertainty and unpredictability linked to the entry into a new productive sector.

### 3.4 Covariates

We also estimate models conditional on a range of demographic and economic covariates. These include age, a binary variable equal to one if the (female) respondent is the head of the household, a binary variable equal to one if she is literate, a binary variable equal to one if she ever attended school, a binary equal to one if she has an infant child (under 12 months of age), household size, the dependency ratio in the household, a binary variable equal to one if the household is polygynous, total per-capita daily expenditure in the household, the household food insecurity access scale, and village fixed effects. The covariates examined correspond to variables that have identified in the literature as predictive of maternal mental health, though it should be noted we do not have data on other potential predictive variables explored in similar analyses – e.g., past history of depression, obstetric complications, and whether or not the pregnancy was unwanted (Alshikh Ahmad et al., 2021; Klainin and Arthur, 2009; Norhayati et al., 2015). Village fixed effects are included to control for other determinants of mental health status that may systematically vary at the local level.^2^

### 3.5 Analysis

The empirical strategy used for the main analysis is relatively simple. In the first cross-sectional analysis, the outcome of interest Y_ict_ for individual i in commune c in year t, corresponding to a measure of stress or post-partum depression, is regressed on a variable capturing empowerment (again, either the continuous empowerment score or a binary variable for empowered). Only data from the baseline round is employed. A linear regression model is employed, and standard errors are clustered at the commune level. We employ a linear model given that we are estimating equations including both binary and continuous dependent variables (Hellevik, 2009). All models are estimated in Stata Version 17.

Y_ict_ ​= ​β X_ict_ ​+ ​ε_ict_

For this initial specification, we also report q-values robust to correction for multiple hypothesis testing (Simes, 1986). We will also estimate a parallel specification conditional on a range of covariates, as described above.

In the second, panel specification, we employ data from both the baseline and follow-up rounds and estimate a parallel specification including individual fixed effects μ_i._ This specification allows us to identify the relationship between changes over time in empowerment status and changes over time in women's well-being.

Y_ict_ ​= ​β X_ict+_μ_i_ ​+ ​ε_ict_

Given the use of individual fixed effects, this specification abstracts from time-invariant cross-sectional variation in both observable and unobservable characteristics that may be correlated with empowerment and mental health. For example, women characterized by better physical health may have systematically higher levels of both empowerment and mental health (and particularly, lower levels of postpartum depression, if better physical health reduces the probability of pregnancy or delivery-related complications), while women characterized by a history of trauma may score lower on both dimensions; neither of these covariates are explicitly measured in this data. In both cases, the unobservable characteristic would serve to bias the estimated coefficient away from zero and lead to an overrejection of the null hypothesis.

The intracluster correlations indicate that within-respondent correlation in the key variables of interest is relatively low: the ICC is between 0.1 and 0.15 for the empowerment and stress scores, and only 0.03 for the depression score. While this suggests ex ante that the inclusion of individual fixed effects may not have substantial effects on the coefficients of interest, it remains useful to assess the robustness of cross-sectional correlations in the panel specification. We also estimate the panel specification including additional time-varying covariates as controls, including a binary variable for whether an infant is present, household expenditure, the HFIAS score, and treatment assignment in the SELEVER trial.

It is important to note that the main trial results suggest that there was generally no significant effect of the intervention on poultry-related outcomes, other than weak evidence of increased use of poultry inputs (Leight et al., 2022); no significant effect on women's empowerment (Heckert et al., 2021); and no significant effect on maternal child and nutrition outcomes (Becquey et al., 2022). Accordingly, while the panel specification does include an additional control for treatment assignment (coded as zero for all households at baseline), previous evidence has already suggested there is no significant correlation between treatment assignment and empowerment.

## 4 Empirical results

### 4.1 Summary statistics

Table 1 presents summary statistics for basic demographic household characteristics (reported at baseline) and variables capturing empowerment and mental health (reported at baseline and endline). The average household includes eight members, and the household heads are characterized by an average age of 33 and a low level of education (only 8% completed primary education). 38% of households are polygynous. Average per-capita household expenditure is only around $.50.

**Table 1:** Summary statistics.

	Baseline			Endline		
	Mean	St. dev.	Obs.	Mean	St. dev.	Obs.
Household size	7.77	3.94	1424			
Age	31.04	7.30	1424			
Male head	0.08	0.16	1424			
Head completed primary education	0.08	0.27	1424	0.07	0.26	1503
Polygynous household	0.38	0.49	1424	0.46	0.50	1503
Total per-capita expenditure daily	0.55	0.61	1420	0.44	0.50	1502
Empowerment score	0.51	0.16	1203	0.51	0.18	1389
Empowered (binary)	0.10	0.30	1203	0.14	0.34	1389
Autonomy in income	0.58	0.49	1424	0.44	0.50	1505
Self-efficacy	0.45	0.50	1424	0.54	0.50	1505
Attitudes about domestic violence	0.53	0.50	1424	0.52	0.50	1505
Respect among household members	0.67	0.47	1385	0.60	0.49	1389
Input in productive decisions	0.80	0.40	1424	0.77	0.42	1505
Ownership of land and other assets	0.82	0.38	1424	0.87	0.34	1505
Access to and decisions on financial services	0.20	0.40	1424	0.21	0.41	1505
Control over use of income	0.67	0.47	1424	0.61	0.49	1505
Work balance	0.26	0.44	1240	0.30	0.46	1505
Visiting important locations	0.54	0.50	1424	0.46	0.50	1505
Group membership	0.34	0.47	1424	0.47	0.50	1505
Membership in influential groups	0.27	0.44	1424	0.39	0.49	1505
Stress score	3.59	3.95	1424	3.28	3.34	1506
Binary variable for high stress	0.34	0.47	1424	0.20	0.40	1506
Depressive symptoms score	5.71	4.76	1424	7.13	5.38	1506
Binary variable for postpartum depression	0.11	0.32	1424	0.19	0.39	1506

Notes: This table reports summary statistics on demographic characteristics, empowerment, and mental health at baseline and endline.

The continuous empowerment score, capturing the share of 12 total domains in which a woman is empowered, is around 0.5 ​at both baseline and endline. Using the binary variable for empowered (again, equal to one if an individual is empowered in nine out of twelve domains), 10% of women are identified as empowered at baseline, and 14% at endline. In general, the level of empowerment observed in this population is somewhat lower vis-à-vis other populations examined: a summary provided in the pro-WEAI overview paper reported an empowerment rate of 16% for women in a pooled sample including five country-specific samples: the SELEVER sample and four others, three collected in Bangladesh and one in Mali (Malapit et al., 2019).

When we examine the indicators that comprise the WEAI, we observe a heterogeneous pattern of shifts from baseline to endline. For some indicators (autonomy in income, respect among household members, control over use of income, and visiting important locations), there are negative shifts over time. For some indicators, there are substantial positive shifts (self-efficacy, group membership, and membership in influential groups). The other indicators show largely stable patterns.

Finally, we report summary statistics for the measures of well-being and mental health. Using the continuous stress scale, the mean score at baseline is around 3.5 on a scale from zero to 20, and this declines slightly from baseline to endline. The probability of a woman identified as experiencing a high level of stress (defined as a SRQ-20 score greater than or equal to five) is 34% at baseline, declining to 20% at endline. Using the Edinburgh scale for post-partum depression, the average level of depressive symptoms is around six at baseline, rising to seven at endline. The probability that a woman is identified as depressed using the validated cutoff of 12 is 11% at baseline, rising to 19% at endline.

### 4.2 Primary analysis

Table 2 reports the simple cross-sectional relationships between empowerment variables (both the continuous empowerment score, and the empowered binary variable) and post-partum depression and stress (again, employing both continuous and binary variables). In Columns (1) through (4), we observe that there is generally a negative association between the empowerment score and the dependent variables capturing stress and post-partum depression, suggestive of a lower incidence of these challenges among women characterized by a higher level of empowerment. However, the estimated coefficients for stress (reported in Columns (1) and (2)) are not statistically significant. The magnitude suggests that a one standard deviation increase in the empowerment score^3^ is associated with a 3 percentage point decline in the probability of a woman being identified as above the threshold for post-partum depression. When using the binary variable for empowered, we observe a similar pattern. The q-values suggest that the primary findings are robust to correction for multiple hypothesis testing.

**Table 2:** Cross-sectional correlations.

	Stress score	Binary variable for high stress	Depressive symptoms score	Binary variable for postpartum depression	Stress score	Binary variable for high stress	Depressive symptoms score	Binary variable for postpartum depression
Empowerment score	−1.398	−0.155	−2.582∗∗∗	−0.194∗∗∗				
	(0.914)	(0.104)	(0.758)	(0.0489)				
q-value	[0.150]	[0.150]	[0.001]	[0.000]				

Empowered (binary)					−0.285	−0.0466	−0.659∗	−0.0843∗∗∗
					(0.334)	(0.0403)	(0.366)	(0.022)
q-value					[0.389]	[0.259]	[0.092]	[0.000]

Observations	1203	1203	1203	1203	1203	1203	1203	1203
R-squared	0.003	0.003	0.007	0.009	0	0.001	0.002	0.006

Robust standard errors in parentheses								

∗∗∗p ​< ​0.01, ∗∗p ​< ​0.05, ∗p ​< ​0.1.

Note: This table reports the correlations between the continuous empowerment score and the binary variable for empowerment, and continuous and binary variables for both stress and depression. Each cell reports the regression coefficient on top, followed by the standard error in parentheses.

Table 3 then reports parallel results conditional on a range of covariates, including village fixed effects. The primary estimated coefficients for the empowerment score and the binary variable for empowered remain similar in magnitude and significance vis-à-vis the results reported in Table 2. A binary variable for ever attending education is associated with a higher depression score, while the presence of an infant is associated with lower stress and depression scores. No other household-level characteristics are significantly associated with stress or depression. (If the same specification is estimated excluding the village fixed effects, however, women in households characterized by a higher HFIAS score, or who are experiencing more food insecurity, report significantly higher levels of both stress and depression.)

**Table 3:** Cross-sectional correlations conditional on additional demographic characteristic.

	Stress score	Binary variable for high stress	Depressive symptoms score	Binary variable for postpartum depression	Stress score	Binary variable for high stress	Depressive symptoms score	Binary variable for postpartum depression
Empowerment score	−0.302	−0.0249	−1.695∗	−0.163∗∗				
	(0.822)	(0.0936)	(0.891)	(0.0615)				
Empowered (binary)					−0.292	−0.0448	−0.662∗	−0.0801∗∗∗
					(0.383)	(0.0452)	(0.386)	(0.0262)
Age	0.0284	0.00131	0.0389	0.00153	0.0285	0.00141	0.0360	0.00132
	(0.0172)	(0.00234)	(0.0247)	(0.00169)	(0.0173)	(0.00233)	(0.0247)	(0.00168)
Female head	0.0984	−0.0495	0.328	0.144	0.0557	−0.0549	0.178	0.128
	(1.046)	(0.100)	(0.996)	(0.0905)	(1.060)	(0.101)	(1.017)	(0.0955)
Literate	−0.152	0.0411	−0.442	−0.0893	−0.136	0.0432	−0.395	−0.0841
	(0.551)	(0.0570)	(0.746)	(0.0538)	(0.550)	(0.0567)	(0.734)	(0.0525)
Ever attended school	0.512	0.0537	0.784∗∗	0.0191	0.510	0.0536	0.776∗∗	0.0183
	(0.377)	(0.0418)	(0.376)	(0.0288)	(0.377)	(0.0421)	(0.376)	(0.0285)
Infant present	−0.661∗∗	−0.0509	−0.623∗	−0.0316	−0.666∗∗	−0.0518	−0.627∗	−0.0323
	(0.274)	(0.0340)	(0.347)	(0.0229)	(0.274)	(0.0341)	(0.347)	(0.0229)
Household size	−0.0434	−0.00593	−0.00382	0.00325	−0.0436	−0.00597	−0.00337	0.00327
	(0.0300)	(0.00400)	(0.0528)	(0.00339)	(0.0297)	(0.00398)	(0.0526)	(0.00330)
Dependency ratio	0.0670	−0.00487	−0.0874	−0.00550	0.0662	−0.00524	−0.0778	−0.00482
	(0.237)	(0.0278)	(0.238)	(0.0148)	(0.237)	(0.0280)	(0.239)	(0.0150)
Polygamous	−0.485	−0.0372	0.0534	−0.00152	−0.494	−0.0386	0.0311	−0.00415
	(0.297)	(0.0371)	(0.446)	(0.0319)	(0.297)	(0.0373)	(0.445)	(0.0317)
Total per-capita expenditure daily	0.0422	0.00997	0.303	0.00276	0.0449	0.0102	0.318	0.00417
	(0.179)	(0.0224)	(0.206)	(0.0167)	(0.179)	(0.0224)	(0.207)	(0.0167)
HFIAS Score (0–27)	0.0284	0.00131	0.0389	0.00153	0.0285	0.00141	0.0360	0.00132
	−0.0256	−0.00328	−0.0538	−0.00306	−0.0254	−0.00325	−0.054	−0.00305

Notes: This table reports the conditional correlations between the continuous empowerment score and the binary variable for empowerment, and continuous and binary variables for both stress and depression, controlling for a range of demographic and economic covariates. Standard errors are clustered at the commune level.

Each cell reports the regression coefficient on top, followed by the standard error in parentheses.

Table 4 reports the panel specification, conditional on individual fixed effects. We observe that the results are both (generally) larger in magnitude and more precisely estimated: a one standard deviation increase in the empowerment score is now associated with a 4.4 percentage point decline in the probability of high maternal stress, and a 2.5 percentage point decline in the probability of post-partum depression. There are also significant correlations between the continuous empowerment score and the continuous measures of stress and depression: a one standard deviation increase in the empowerment score is associated with a 0.11 standard deviation decline in both the stress and depression scores. Using the binary variable for empowered, only the correlations with high maternal stress are statistically significant, suggesting that women who are empowered experience a 0.2 standard deviation decline in the reported stress score, and are 10 percentage points less likely to be characterized by high maternal stress. Panel B reports the same relationships conditional on time-varying covariates, and the patterns of magnitude and significance again remain consistent. (Time-invariant characteristics are not included in this specification given that they are collinear with individual fixed effects.)

**Table 4:** Panel correlations.

	Stress score	Binary variable for high stress	Depressive symptoms score	Binary variable for postpartum depression	Stress score	Binary variable for high stress	Depressive symptoms score	Binary variable for postpartum depression
Panel A: Simple panel correlates								
Empowerment score	−2.620∗∗∗	−0.268∗∗∗	−3.495∗∗∗	−0.165∗∗				
	(0.777)	(0.0914)	(1.162)	(0.0819)				
Empowered (binary)					−0.787∗∗	−0.0926∗∗	−0.616	−0.0231
					(0.360)	(0.0396)	(0.526)	(0.0405)

Observations	2020	2020	2020	2020	2020	2020	2020	2020
R-squared	0.014	0.009	0.013	0.006	0.006	0.005	0.002	0.001

Panel B: Estimates conditional on time-varying covariates								
Empowerment score					−0.749∗∗	−0.0684∗	−0.688	−0.0191
					(0.333)	(0.0381)	(0.499)	(0.0386)
Empowered (binary)	−2.549∗∗∗	−0.258∗∗∗	−3.401∗∗∗	−0.158∗∗				
	(0.744)	(0.0906)	(1.134)	(0.0796)				

Observations	2014	2014	2014	2014	2014	2014	2014	2014
R-squared	0.056	0.025	0.033	0.014	0.05	0.022	0.023	0.009

Robust standard errors in parentheses								
∗∗∗p ​< ​0.01, ∗∗p ​< ​0.05, ∗p ​< ​0.1								

Note: This table reports the correlations between the continuous empowerment score and the binary variable for empowerment, and continuous and binary variables for both stress and depression, in a panel specification controlling for individual fixed effects. Standard errors are clustered at the commune level. Each cell reports the regression coefficient on top, followed by the standard error in parentheses.

Finally, Table 5 reports comparable results in which we analyze the predictive effects of each pro-WEAI indicator, using the panel specification conditional on individual fixed effects and time-varying covariates. In general, the evidence suggests that self-efficacy and respect among household members are the most strongly predictive of decreased incidence of stress and post-partum depression; these coefficients are large in magnitude and precisely estimated. Women characterized by high self-efficacy show a six percentage point decline in the probability of high maternal stress. Women reporting respect among household members show a 11 percentage point decline in the probability of high maternal stress, and a five percentage point decline in the probability of post-partum depression. Autonomy in income is also predictive of lower depression and stress, and there is some evidence that input in productive decisions is associated with lower stress.

**Table 5:** Panel correlations by WEAI indicator.

	Stress score	Binary variable for high stress	Depressive symptoms score	Binary variable for postpartum depression
Autonomy in income	−0.495∗∗	−0.0192	−1.001∗∗∗	−0.0727∗∗∗
	(0.219)	(0.0266)	(0.346)	(0.0216)
Self-efficacy	−0.602∗∗	−0.0596∗	−0.987∗∗∗	−0.0320
	(0.277)	(0.0336)	(0.354)	(0.0251)
Attitudes about domestic violence	−0.228	−0.0403∗	−0.176	−0.0129
	(0.192)	(0.0237)	(0.295)	(0.0212)
Respect among household members	−0.898∗∗∗	−0.108∗∗∗	−0.765∗∗	−0.0461∗∗
	(0.206)	(0.0273)	(0.361)	(0.0209)
Input in productive decisions	−0.726∗∗∗	−0.0423	−0.527	−0.0242
	(0.274)	(0.0353)	(0.327)	(0.0293)
Ownership of land and other assets	0.0787	−0.0129	0.638	−0.0119
	(0.330)	(0.0431)	(0.441)	(0.0315)
Access to and decisions on financial services	0.721∗∗	0.0315	1.848∗∗∗	0.111∗∗∗
	(0.312)	(0.0374)	(0.440)	(0.0315)
Control over use of income	0.347	0.0164	−0.492	−0.0157
	(0.238)	(0.0307)	(0.355)	(0.0281)
Work balance	0.216	0.0254	−0.0488	−0.0227
	(0.233)	(0.0282)	(0.416)	(0.0311)
Visiting important locations	−0.0781	0.00870	0.150	0.0180
	(0.218)	(0.0271)	(0.292)	(0.0211)
Group membership	−0.0653	−0.0642	0.508	0.0629
	(0.423)	(0.0579)	(0.691)	(0.0399)
Membership in influential groups	−0.263	0.0355	−1.102	−0.0801∗
	(0.428)	(0.0565)	(0.757)	(0.0428)

Note: This table reports the correlations between the component WEAI indicators and continuous and binary variables for both stress and depression, in a panel specification controlling for individual fixed effects. Standard errors are clustered at the commune level. Each cell reports the regression coefficient on top, followed by the standard error in parentheses.

By contrast, access to and participation in decisions around financial services seems to be associated with higher depression and stress. The remaining indicators are generally statistically insignificant, including attitudes around domestic violence, ownership of land and other assets, control over and use of income, work balance, visiting important locations, group membership, and membership in influential groups.

## 5 Discussion

The results presented in this paper suggest that women's empowerment is a significant predictor of both maternal stress and depressive symptoms in a large sample of rural women in Burkina Faso, using both cross-sectional and panel specifications. Broadly, our findings are consistent with the existing literature on empowerment and mental health. In Senegal, empowerment (measured using a series of binary questions about household decision-making) is negatively associated with self-reported anxiety (Fielding and Lepine, 2017). Another recent paper performs a similar analysis in Laos and finds similar patterns, though self-reported well-being is measured using a Likert scale of satisfaction (Yoshikawa and Kamiya, 2019). In Egypt, empowerment enabling resources (measured as higher schooling attainment, premarital economic activity, later age at first marriage, and greater proximity to natal family) have a negative effect on generalized anxiety (Yount et al., 2014). In India, more decision-making power is identified as a protective factor vis-à-vis common mental disorders (measured using the SRQ) in a large cross-sectional survey of women (Scott et al., 2020).

Our findings of a negative association between empowerment and stress and depressive symptoms are consistent with this existing body of literature, but also build on it in several respects. We demonstrate that this association holds even in a longitudinal analysis conditional on individual fixed effects, a novel finding in the literature, and provide the first evidence from West Africa and Burkina Faso specifically. The analysis also provides valuable evidence pinpointing the specific facets of empowerment that are most predictive of lower stress and depression: particularly, higher self-efficacy and stronger relationships among family members. In comparing the magnitudes of the estimated associations in this paper to previous literature, some differences in estimation strategies render comparisons challenging: the papers from Egypt and India report analyses only of household-level socioeconomic covariates that may be linked to empowerment (Yount et al., 2014), or a continuous empowerment score that is distinct from the pro-WEAI (Scott et al., 2020). However, in Senegal, women coded as empowered using a binary variable capturing freedom of movement are 10 percentage points less likely to experience frequent stress and anxiety (Fielding and Lepine, 2017), a magnitude very similar to the association observed here, where more empowered women are 11 percentage points less likely to report high maternal stress. In Laos, a somewhat different relationship is observed to be larger: women who report autonomy are around 25 percentage points more likely to report high satisfaction (Yoshikawa and Kamiya, 2019), but this may reflect the very different connotations of satisfaction vis-à-vis (absence of) stress.

More broadly, there is some evidence around the determinants of stress and anxiety in low-income countries, though the existing literature largely focuses on post-partum depression. A systematic review of the prevalence and correlates of post-partum depression identified a number of health-related risk factors as well as poor marital relationships, stressful life events, and absence of social support as key predictors of depression (Norhayati et al., 2015). A number of country-specific analyses conducted in sub-Saharan Africa and South Asia as well as additional regional systematic reviews focused on South Asia and Middle East present similar evidence, but do not analyze the full range of socioeconomic and empowerment-related covariates analyzed here (Abrahams and Stellenberg, 2015; Adewuya et al., 2007; Alshikh Ahmad et al., 2021; Atuhaire et al., 2021; Azad et al., 2019; Gausia et al., 2009; Klainin and Arthur, 2009; Rahman et al., 2003; Wubetu et al., 2020). The absence of social support is frequently identified as a major correlate of postpartum depression risk, and some papers analyze rurality: e.g., there is evidence of a higher risk of post-partum depression for rural women in Uganda (Atuhaire et al., 2020). Results linked to poverty or socioeconomic status are mixed: poverty is not significantly correlated with postpartum depression in Bangladesh (Azad et al., 2019), but this correlation is observed in the Middle East (Alshikh Ahmad et al., 2021).

Here, we find that there is no evidence of any correlation between socioeconomic characteristics and mental health, conditional on village fixed effects. Women who report any education are characterized by a higher level of anxiety and depression, while women reporting a child under one in their household (as distinct from older children) are characterized by a lower level of anxiety and depression. Both of these latter findings are novel, and may be surprising, though these patterns could reflect the fact that women who are more educated may experience a sense of separation from their peers and the associated social network, while women with infants may be more connected to social networks. The literature on post-partum depression generally emphasizes the risks in the immediate post-partum period, though there has been little systematic examination of the relationship between time elapsed since birth and the incidence of postpartum depression in LMICs.

In addition, the detailed analysis of pro-WEAI components suggests that variables linked to self-efficacy and autonomy are highly predictive of lower depression and anxiety, a finding that is new in the literature focusing on low-income countries. Respect among household members is also strongly predictive of lower depression and anxiety, building on existing literature suggesting that higher social support reduces the risk of mental health challenges.

(Adewuya et al., 2007; Myer et al., 2008; Norhayati et al., 2015; Qadir et al., 2013; Rungreangkulkij et al., 2019; Thomas, 2006). Interestingly, the pro-WEAI indicators in the domain of instrumental agency – capturing control over resources of various types – are largely not meaningfully associated with enhanced mental health. This includes input into productive decisions; ownership of land and other assets; access to and decisions on credit; control over use of income; work balance; and visiting important locations. In particular, access to and decisions around credit are significantly associated with higher levels of depression.

In the previous literature, there is in fact some evidence that loan access can have adverse effects on stress and/or mental health in South Africa, though the patterns of effects by gender varies (Fernald et al., 2008; Karlan and Zinman, 2010). An analysis in Bangladesh finds null effects of microcredit on mental health (Masud Ahmed et al., 2001), while there is evidence of mental health benefits of financial inclusion in Nigeria (Ajefu et al., 2020). One hypothesis that would be consistent with the observed pattern here is that participating in decisions around or accessing credit significantly increases women's self-perceived burden of responsibility linked to economic decision-making in the household and thus worsens their mental health. This may be a fruitful area for future research.

There are two important challenges to note in interpreting the association between the pro-WEAI index and mental health presented here. First, in a household bargaining framework, women's welfare outcomes would also be meaningfully shaped by relative power: the level of decision-making power that a woman possesses vis-à-vis her spouse, or other key decision-makers. The pro-WEAI index generally abstracts from capturing this dynamic in which one spouse (often, though not always, the man) may have power over his or her partner or decisions that crucially affect the partner, though it does capture input into household decision-making more broadly. That being said, a woman who is identified as empowered in the pro-WEAI could still be characterized as having lower bargaining power than her husband in a more traditional analysis of intrahousehold dynamics. Second, the pro-WEAI is constructed as an equally weighted index comprised of a broad set of different indicators, potentially rendering interpretation challenging. There is some evidence in the literature, however, that empowerment rankings across different samples using the pro-WEAI are robust to alternate weightings (Malapit et al., 2019).

This analysis has a number of strengths and limitations. We employ a dataset that includes rich and detailed measures of multiple dimensions of both mental health and empowerment, and are able to use the panel structure of the data to control for time-invariant individual-level observable and unobservable characteristics.

Key limitations in measurement include the fact that the sample includes women who are not immediately post-partum; the use of the SRQ-20 to measure stress rather than a more focused indicator such as the Perceived Stress Scale; and the absence of data capturing other dimensions of mental health (e.g., anxiety). In addition, there is no random or quasi-random variation in empowerment that would allow us to better assess causality. This associational analysis renders interpretation of the relationship between empowerment and maternal health more challenging and suggests the findings may not be fully generalizable, especially in very different economic or cultural contexts.

To sum up, the evidence provided in this paper is suggestive of another important pathway through which women's empowerment may shape women's welfare: enhanced mental health. In addition, given the substantial evidence that challenges linked to maternal health are associated with adverse nutritional and health outcomes for infants and children in low-income countries (Bennett et al., 2016; Harpham et al., 2005; Nguyen et al., 2014; Patel et al., 2004), the relationship between empowerment and mental health also has meaningful implications for children's welfare.

## Funding

We acknowledge funding support from the 10.13039/100000865Bill & Melinda Gates Foundation (grant no. OPP1149709) and the 10.13039/501100015815CGIAR Research Program on Agriculture for Nutrition and Health (A4NH), led by IFPRI (sub-grant no. A4NH- 202004.040.500).

## CRediT authorship contribution statement

**Jessica Leight:** formulated the research question for this paper, conducted the analysis, and drafted the manuscript. **Abdoulaye Pedehombga:** led in managing the data collection, with support. **Rasmané Ganaba:** led in managing the data collection, with support. **Aulo Gelli:** led in the overall design of the study and the management of the data collection.

## Declaration of competing interest

The authors declare that they have no known competing financial interests or personal relationships that could have appeared to influence the work reported in this paper.

## Footnotes

1 We do not impose any quality criteria for evidence included in this overview; rather, the main criteria employed is that the paper report empirical evidence from a low-income or middle-income country around the relationship between a clearly defined variable capturing the channel of interest and a well-specified index measuring mental health.

2 Estimating the same specification without the inclusion of village fixed effects in the controls yields extremely similar findings.

3 The standard deviation of the empowerment score in this sample is .166.
