# Supplementary material for: Women's empowerment, maternal depression, and stress: Evidence from rural Burkina Faso
Source: SSM Ment Health. 2022 Dec;2:100160. doi: 10.1016/j.ssmmh.2022.100160 (PMC9792374; doi:10.1016/j.ssmmh.2022.100160)
Supplement: Multimedia component 1 [file mmc1.docx]

Supplementary material

The SELEVER intervention is multifaceted, encompassing intervention components in three broad sectoral areas: poultry, nutrition and health, and gender.

The poultry intervention centered around the provision of training around poultry production to rural households constituted in village-level producers' and savings groups. Eight poultry training modules, delivered in up to eight sessions, were delivered by trained facilitators (similar to extension agents) to the producers' groups. While the precise mechanism of delivery varied locally, a process evaluation conducted by the research team suggested that frequent modes of delivery included initial workshops of longer duration (two to three days) in conjunction with more regular training sessions, delivered by screening video training modules, that were offered in conjunction with producer associations' regularly scheduled (approximately monthly) meetings.

In addition, SELEVER sought to strengthen village animal health and credit services. Rural poultry producers rely on a system of Village Volunteer Extension Services Workers (VVVs following the name in French), a volunteer corps trained by government staff to provide basic livestock services (e.g., vaccinations, deworming and nutrition advice). Importantly, VVVs are active across the sample in both treatment and control arms, and they are not an innovation introduced by this intervention. However, SELEVER provided additional training to the VVVs as well as a start-up kit including a cold storage unit, a syringe and needle, and 100 doses each of poultry vaccines and deworming pills. The program also developed a cohort of women VVVs to better reach women poultry producers.

Finally, in the credit sphere, two microcredit organizations collaborated with Tanager to expand into the treatment communes, providing at least one microcredit branch in each commune if such a branch was not already present. They also assumed the role of providing credit to poultry producers participating in SELEVER, but there was no explicit guarantee. Credit was available based on standard commercial criteria designed to identify a viable enterprise, and these criteria were largely discretionary.

The nutrition component of SELEVER included BCC on nutrition and diets provided through women’s groups, poultry producer groups, and local community leaders. The topics of the BCC activities focused on improved diets at key stages of the life cycle: this included IYCF practices and diet diversity promotion through the promotion of daily consumption of three food groups: energy-giving foods, protective foods, and body-building foods.

Finally, the gender component included community-level sensitization on women’s economic empowerment and gender equity, including strengthening of women’s groups, training participants from existing women’s associations on enterprise development, and strengthening women’s role in decision-making within households and in the community.

Table S1 summarizes some information about program participation. In general, it is evident that participation in SELEVER program elements was not particularly high: around 25% of households in communities assigned to the treatment arm participated in trainings, and around 5% reported participation in community groups focused on gender or nutrition. There is some evidence of program spillovers in control communities, though the rate of spillover is not high. Households are more likely to report receipt of VVV services, but this is only slightly higher in treatment communities vis-à-vis control communities.

Table S1: Participation in SELEVER

|  | Mean in control communities | Mean in treatment communities |
| --- | --- | --- |
| Participated in a poultry training | 0.07 | 0.27 |
| Participated in a gender or nutrition training | 0.12 | 0.24 |
| Member of a nutrition-focused community group | 0.03 | 0.06 |
| Member of a gender-focused community group | 0.01 | 0.05 |
| Received VVV services | 0.54 | 0.59 |
